# Endometriosis in Adolescence

**DOI:** 10.1155/2012/869191

**Published:** 2012-10-10

**Authors:** Margherita Dessole, Gian Benedetto Melis, Stefano Angioni

**Affiliations:** Division of Gynecology and Obstetrics, Department of Surgical Science, University of Cagliari, Via Ospedale, 09124 Cagliari, Italy

## Abstract

Endometriosis is a common cause of pelvic pain and infertility. The majority of women report symptoms since adolescence, and there are rare cases of endometriosis in premenarchal age patients. Symptoms in adolescence are similar to those in adulthood. Treatment usually consists of oral contraceptives and nonsteroidal anti-inflammatory drugs. In cases where this treatment is not successful, laparoscopy and biopsy of the lesions are necessary for diagnosis. However, emerging new technologies provide new options, in particular the use of serological markers.

## 1. Introduction

Endometriosis is a common benign and chronic gynaecologic disorder related to the presence of endometrial glands and stroma outside of their normal location [1]. The disease often begins in adolescence, but is most often recognized after years of dysmenorrhea. Thanks to increased availability of modern imaging techniques, such as ultrasound and MRI, it is now possible to provide earlier detection.

## 2. Epidemiology

Endometriosis is the most common cause of secondary dysmenorrhea in adolescence [2]. The prevalence of endometriosis in the general population is estimated to be between 0.7 and 44%. The true incidence of endometriosis in adolescents is difficult to quantify and estimates vary among different studies [3]. Goldstein et al. [4] reported a 47% incidence of disease in adolescent girls undergoing laparoscopy for chronic pelvic pain, whereas Laufer et al. [5] reported a finding of endometriosis at the time of surgery in 67% of adolescents who have pain refractory to common medical treatments like nonsteroidal anti-inflammatory agents (NSAIDs) or oral contraceptive pills (OCPs). Although in the past it was assumed that endometriosis presented only after many years of menstruation, studies have shown endometriosis to occur prior to menarche [6] and between 1–6 [7, 8] months after the onset of menarche. According to The Endometriosis Association, 66% of adult women with endometriosis report the onset of pelvic symptoms prior to age 20. Most importantly, patients who report symptoms as teens visit an average of 4 or more physicians before receiving a correct diagnosis [3, 9].

## 3. Etiology

Many theories have been proposed to explain the pathophysiology of endometriosis, leading the name of the “disease of theories.” In 1927 Sampson suggested that retrograde menstruation through the fallopian tubes results in seeding the pelvis with endometrial tissue. These endometrial cells implant themselves on the peritoneal surface of abdominal and pelvic organs and tissues. With consecutive menstrual cycles, they undergo proliferation and bleeding, with the possibility of causing further disseminations and implants [10]. This theory does not explain the presence of disease in women with Mullerian agenesis, aplasia, or endometriosis before menarche [4, 5, 8], with or without obstructive anomalies. The latter could be explained by the coelomic metaplasia theory postulated in 1919 by Meyer, which assumed that metaplasia of the multipotential coelomic epithelium is the origin of endometriosis, as peritoneal and endometrial cells are both derived from the same embryonic precursor. The presence of stress, such as inflammation or irritation (and/or abnormal estrogen stimulation) from refluxed menstrual tissues, causes the coelomic cell, previously differentiated into peritoneal cell, to transform into endometrial cells, which respond in cyclic manner. This theory not only explains the presence endometriosis in all of the possible anatomical sites, but also justifies the presence of disease in women without a uterus, in very young pre-menarchal girls and even in males.

Other theories worthy of note include that of Halban (1924) which identifies endometrial lesions in the extra pelvic endometrial cells which have penetrated the lymphatic and blood vessels as being responsible for the process of embolization, often in ectopic site. The theory of the hormonal milieu assumes that the disease depends on the presence of circulating steroid hormones. And finally the theory of immunity by Weed and Arquenborg (1980) which links the disease to immune alterations that favour the onset of lesions in the peritoneal cavity after the process of retrograde menstruation described by Sampson.

## 4. Symptoms

In adolescents, the symptoms of endometriosis are  chronic pelvic pain (often acyclical), while symptoms in adult women are cyclical chronic pelvic pain, progressive worsening dysmenorrhea, and dyspareunia, in cases where they are sexually active [11].

Bowel and bladder symptoms are also common in adolescents [5]. The localization in the ovary (ovarian endometrioma) is rare before 25 years. Some authors report that acyclic chronic pelvic pain and a nonresponding of pharmacological treatment with OCPs and NSAIDs in adolescence are sufficient to warrant diagnostic laparoscopy [12].

## 5. Diagnosis

There are a variety of methods that can be used to assess whether a woman has endometriosis, but the only reliable way to confirm the disease is by visually inspecting the abdominal organs and biopsy using laparoscopic methods. This leads to the majority of women with chronic pelvic pain to prefer empirical treatment prior to choosing surgery Thanks to modern imaging techniques such as ultrasound and MRI, it is now possible to make a less invasive diagnosis of endometriosis. The diagnostic procedure does not differ in adolescents or adults, but a careful history is crucial in young women to determinate the chronicity of the pain, related bowel or bladder problems, and the responsiveness to drugs. M. R. Laufer et al. [13] suggested a pain diary, where the patient describes and documents the frequency and character of the latter. Adequate family history is essential information, as the incidence of endometriosis in patients with affected family members is 6.9% compared to 1%-2% for the general population [14].

A physical exam is very important, but it may not be available for all adolescents. The goal of the physical exam should be to try to determine the etiology of the pain and exclude all other causes.

For an adolescent who is not sexually active, a rectal abdominal exam can be performed. A common finding on pelvic exam in these patients includes clu-de-sac tenderness [15].

Pelvic ultrasound remains a cornerstone in the diagnosis of this disease, although it is less helpful in adolescents as the endometrioma is rare in adolescents. An MRI examination is a better diagnostic tool, but the elevated cost makes it less accessible. Endometrial biopsy with assessment of the amount of nerve fibres has been recently reported to be a successful approach. According to a recent study, the specificity for endometriosis of the endometrial biopsy with immunohistochemical analysis of nerve fibres was detected by 83% and sensitivity of 98% with a positive predictive value of 91% and a negative predictive value of 96%. A second study by the same author showed the endometrial density of the nerve fibres to be approximately 14 times greater in women with endometriosis. Immunohistochemical analysis allowed for the localization of neural markers for sensory C, A delta, adrenergic, and cholinergic nerve functional layer of the endometrium. Sections were immunostained with PGP9.5, anti-neurofilament protein, SP, VIP, anti-neuropeptide Y, and anticalcitonine gene-related polypeptide.

Sensitivity for minimal to mild endometriosis was 95% with use of combined analysis of neural markers PGP9.5, VIP, and SP. Specificity was 100% and accuracy was 97.5% [16]. The endometrial biopsy is performed with hysteroscopy with a vaginoscopic approach [17]. This method could be utilised for adolescents with suspected endometriosis. 

The biochemical markers of this disease have been known for years, and new developments may permit a noninvasive and timely diagnosis. CA 125, Ca 19.9, ICAM-1, and IL-6 together with follistatin and urocortin have proven to be the most reliable markers for endometriosis diagnosis [18].

CA 125 (nv < 35 IU/mL) is a member of the mucin family glycoproteins and is a tumor marker or biomarker for patients with endometrium, ovary, breast, lung, and gastrointestinal cancers. Reports show that the serum level of CA 125 increases in the case of endometriosis and that the values tend to increase in the more advanced stages of disease (III-IV) compared to the initial stages (I-II). CA 125 takes a better diagnostic significance when used in the assessment of disease recurrence and followup after surgical treatment [19].

CA 19.9, a specific marker for the diagnosis of pancreatic cancer, is elevated in benign conditions such as diseases of the biliary tract and endometriosis. The value of these markers is not influenced by the stage of the disease as with Ca 125 [20].

ICAM-1 is a member of the immunoglobulin superfamily and is involved in immune and inflammatory response. It has been evaluated as a potential new marker of endometriosis. Ectopic endometrial stromal cells have a form of ICAM-1 on the cell surface that is physiologically released in soluble form and detectable in the serum. In the case of endometriosis, greater amounts of ICAM-1 are released by the ectopic endometrium and by the endometriosis implants and found to be significantly higher serum concentrations (nv : 410.4 ng/mL) compared with controls. Higher concentrations have been reported in case of deep endometriosis than peritoneal surface endometriosis [21].

Considering the family of interleukins, patients with endometriosis have higher levels of IL-6. A significant diagnostic accuracy (sensitivity 75.0%, specificity 83.3%) was found for the initial stages of the disease (I-II) using a cutoff of 25.75 pg/mL. 

The combined evaluation of dosages of Ca-125, Ca-19.9, and IL-6, however, does not provide significant diagnostic information with respect to the determination of Ca-125 serum levels alone. On the contrary, the diagnostic performance of the assay of ICAM-1 and Ca-125 increases the sensitivity and specificity by 28% and 92%. Therefore, their simultaneous measurement can increase the specificity in the diagnosis of deep infiltrating endometriosis [22].

Recently, two new molecules urocortin (UCN) and follistatin (FS) are being investigated. UCN is a molecule belonging to the family of corticotrophin-releasing factor (CRF), which is involved in the modulation of the immune system and inflammation, physiologically expressed in endometrium during the menstrual cycle, mainly in the secretory phase. It was showed that UCN concentrations in patients with ovarian endometriosis are higher compared to patients with benign ovarian cysts. Concentrations were twice as high in women with endometrioma compared to the control group with a concentration significantly higher in the cystic fluid from endometriomas rather than in the peritoneal fluid and plasma. Higher concentrations of UCN were found in 88% of cases of endometrioma with a specificity of 90%, while that of Ca-125 increased only in 62% of cases. The measurement of the UCN is a promising marker for early detection of differential endometrioma compared to benign ovarian cysts [23].

Similar results have been achieved through the evaluation of FS, an active A binding protein that inhibits its function, but is usually expressed in the normal endometrium. Recent studies have revealed greater tissue expression of FS in the case of endometriosis and high FS concentrations in the cystic fluid compared to plasma and peritoneal fluid. Raised serum levels in the event of endometrioma were found compared to other benign cystic lesions. FS shows a sensitivity of 92% and a specificity of 96% with a cutoff of 1433 pg/mL, while Ca-125 shows only 44% of endometriomas with a specificity of 90% [24]. 

## 6. Therapies

The first-line therapy in adolescents with endometriosis or where endometriosis is suspected is made by oral contraceptives (OCPs) and analgesics (NSAIDs). Unfortunately many of these patients do not respond to this therapy. The alternative options are either therapy with GnRH analogues (only in patients over 18 years) or laparoscopy. The use of GnRH analogues during adolescence is very controversial, as possible impacts on bone mass have been reported. Some authors evaluate this treatment too “invasive.” A 2007 study by Divasta et al. on 36 adolescents between 13 and 21 years old showed that the use of norethindrone acetate as add-back therapy in adolescents treated with a GnRH agonist for endometriosis improves skeletal health [25].

Laparoscopy remains the fundamental diagnostic tool for endometriosis, in particular when pharmacological treatment is not successful. Operative laparoscopy allows for a definitive diagnosis as well as treatment of endometriosis itself [26, 27]. The operation should be performed by a gynaecologist who is comfortable working with women at such a young age, as well as expert on the disease and its multifocality. A clear understanding of the difference of endometriotic lesions in adolescents compared to those of adult women is needed. In teenagers, red lesions are predominant with atypical or white colour but rarely blue or brown lesions found in older patients [28]. In young patients, surgical treatment alone in these cases is not considered appropriate as microscopic residual disease may persist. Therefore, medication is always recommended after surgical therapy to prevent recurrences [29]. In fact, younger age has been shown as an independent risk factor to endometriosis recurrence after conservative surgical treatment of endometriosis [30].

## 7. Conclusion

An early diagnosis of endometriosis and a prompt treatment reduce the risk of future sequelae including multiple laparoscopy in the adulthood, need for assisted reproduction technologies, and a loss of life quality. In fact, for young patients, this chronic disease could significantly impact social and scholastic conditions. These patients should also receive proper counselling as well as psychological support.
